# Infra-Temporal and Pterygo-Palatine Fossae Tumors: A Frontier in Endoscopic Endonasal Surgery—Description of the Surgical Anatomy of the Approach and Report of Illustrative Cases

**DOI:** 10.3390/ijerph19116413

**Published:** 2022-05-25

**Authors:** Matteo Zoli, Giacomo Sollini, Fulvio Zaccagna, Viscardo Paolo Fabbri, Lorenzo Cirignotta, Arianna Rustici, Federica Guaraldi, Sofia Asioli, Caterina Tonon, Ernesto Pasquini, Diego Mazzatenta

**Affiliations:** 1 Programma Neurochirurgia Ipofisi—Pituitary Unit, IRCCS Istituto delle Scienze Neurologiche di Bologna, 40139 Bologna, Italy; federica.guaraldi@yahoo.it (F.G.); sofia.asioli3@unibo.it (S.A.); diego.mazzatenta@unibo.it (D.M.); 2 Department of Biomedical and Neuromotor Sciences (DIBINEM), University of Bologna, 40125 Bologna, Italy; fulvio.zaccagna@unibo.it (F.Z.); viscardopaolo.fabbr2@unibo.it (V.P.F.); caterina.tonon@unibo.it (C.T.); 3 ENT Unit, Bellaria Hospital, Azienda Unità Sanitaria Locale, 40133 Bologna, Italy; sollinigiacomo@gmail.com (G.S.); ernesto.pasquini@ausl.bologna.it (E.P.); 4 Programma Neuroimmagini Funzionali e Molecolari, IRCCS Istituto delle Scienze Neurologiche di Bologna, 40139 Bologna, Italy; 5 Department of Experimental, Diagnostic and Specialty Medicine (DIMES), University of Bologna, 40138 Bologna, Italy; lorenzo.cirignotta@studio.unibo.it (L.C.); arianna.rustici2@unibo.it (A.R.)

**Keywords:** skull base, endoscopy, pterygopalatine fossa, infratemporal fossa, tumors, neuroradiology, malignancies, meningiomas, schwannomas, surgery

## Abstract

Infratemporal and pterygopalatine fossae (ITF and PPF) represent two complex paramedian skull base areas, which can be defined as jewelry boxes, containing a large number of neurovascular and osteomuscular structures of primary importance. They are in close communication with many craniofacial areas, such as nasal/paranasal sinuses, orbit, middle cranial fossa, and oral cavities. Therefore, they can be involved by tumoral, infective or inflammatory lesions spreading from these spaces. Moreover, they can be the primary site of the development of some primitive tumors. For the deep-seated location of ITF and PPF lesions and their close relationship with the surrounding functional neuro-vascular structures, their surgery represents a challenge. In the last decades, the introduction of the endoscope in skull base surgery has favored the development of an innovative anterior endonasal approach for ITF and PPF tumors: the transmaxillary-pterygoid, which gives a direct and straightforward route for these areas. It has demonstrated that it is effective and safe for the treatment of a large number of benign and malignant neoplasms, located in these fossae, avoiding extensive bone drilling, soft tissue demolition, possibly unaesthetic scars, and reducing the risk of neurological deficits. However, some limits, especially for vascular tumors or lesions with lateral extension, are still present. Based on the experience of our multidisciplinary team, we present our operative technique, surgical indications, and pre- and post-operative management protocol for patients with ITF and PPF tumors.

## 1. Introduction

Endoscopic surgery has undoubtedly improved the management of many skull base tumors in the last two decades [1,2,3,4]. Recently, several studies have outlined the effectiveness of the endoscopic endonasal approach (EEA) also for the treatment of lesions involving two difficult anatomical areas such as infratemporal (ITF) and pterygopalatine (PPF) fossae [1,2,3,4].

Indeed, these fossae are considered among the most challenging spaces for skull base surgeons for the high number of neuro-vascular and muscular structures of primary importance for craniofacial and meninges blood supply, sensory innervation of splanchnocranium, nasal and oral glands secretion, lacrimation, and mastication. Due to their communications with several important craniofacial areas, including the middle cranial fossa, the orbit, and the nasal and oral cavities, ITF and PPF are frequently involved by tumoral, infective, or inflammatory lesions spreading directly from these nearby spaces [1,2,3,4]. Standard access to these deep-seated and highly functional fossae is mainly performed through external lateral routes, named infratemporal (IT) approaches, also known as Fisch approaches [5,6]. Those approaches are usually classified as ITa, Itb, and Itc, and recently, Sekhar et al. proposed an additional one, the preauricular, subtemporal-infratemporal approach or Itd [7]. The ITa is mainly directed toward the jugular foramen, the infra-labyrinthine area, and the upper parapharyngeal retrostyloid space, and thus it is less used for ITF and PPF tumors. Instead, the ITb, ITc, and ITd approaches are extended more anteriorly, from the internal carotid artery to the petro-clival region, providing an excellent and wide exposure of ITF and PPF. Therefore, they are still currently considered the gold standard for lesions of these areas [1,6,8].

However, these approaches require extensive cervical and temporal dissection, petrous bone drilling, and eventually zygomatic bone or mandibular ramus resection. Thus, they are burdened by a significant rate of soft tissue disruption, unaesthetic craniofacial scars, and a consistent risk of cranial nerves injuries [1,6]. To reduce the invasiveness and morbidity of these surgical corridors, alternative routes to ITF and PPF, characterized by the use of an anterior or of a combined anterolateral corridor, have been proposed, as the transmaxillary or transfacial ones [8].

In the last years, the role of these anterior routes has been extensively reconsidered, thanks to the introduction of the endoscope in skull base surgery [9]. Indeed, since the beginning of the 2000s, some pioneeristic anatomical and clinical studies have assessed the feasibility, safety, and effectiveness of a purely endoscopic endonasal route to ITF and PPF tumors [10,11,12,13,14,15,16,17,18]. Indeed, it offers a wide and panoramic exposure of these anatomical structures with a direct and straightforward surgical trajectory [10,11,12,13,14,15,16,17,18]. Although some authors have proposed that EEA could be considered the first choice for many malignant and benign tumors of ITF and PPF, no clear agreement exists about the surgical indications and the limits of this approach [15,16,17,18].

The aim of this article is to describe the anatomical and surgical bases of EEA to ITF and PPF tumors, reporting our surgical technique, indications, and limits, and our pre- and post-operative patient management protocol, illustrated by three exemplificative cases.

## 2. Anatomy of Infratemporal Fossa

### 2.1. Boundaries

**Figure 1:**
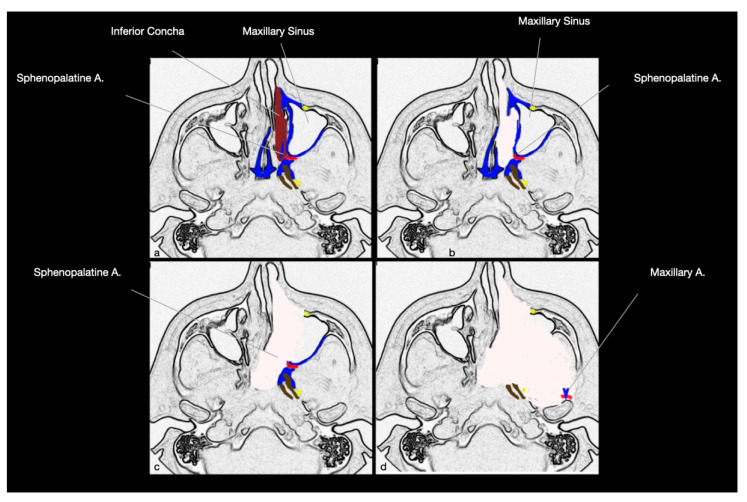
Schematic representation of the different surgical steps for endoscopic transmaxillary-pterygoid approach for ITF and PPF tumors. The anatomical relationship between the posterior wall of maxillary sinus and these fossae is at the base of such approach. (**a**) The inferior nasal concha is completely or partially resected. (**b**) The ostium of maxillary sinus (semilunar hiatus) can be identified. (**c**) An antero-medial maxillectomy, with ipsilateral spheno-ethmoidotomy is performed to expose the posterior wall of maxillary sinus. Sphenopalatine artery is coagulated or litigated after its exposure in the sphenopalatine foramen. (**d**) The posterior wall of maxillary sinus is resected as largely as needed, depending on tumor extension. Maxillary artery is usually clipped to avoid intra- or peri-operative bleedings. (A: Artery).

**Figure 2:**
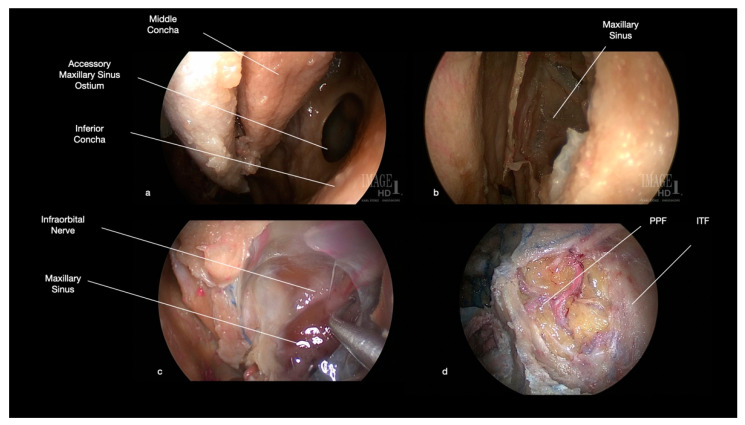
Anatomical endoscopic endonasal dissection (0° degree scope). (**a**) the natural and accessory ostia of the maxillary sinus are identified after resection of the inferior nasal concha and medialization of the middle one. (**b**) The semilunar hiatus is progressively enlarged to perform a medial maxillectomy. The frontal process of the maxilla is resected as much as necessary to achieve a sufficient exposure of the lateral part of the posterior wall of the maxillary sinus. (**c**) The most relevant anatomical structure in the superior wall of the maxillary sinus is represented by the infra-orbital nerve. (**d**) After resection of the posterior wall of maxillary sinus and opening of the periosteum, access to ITF and PPF is realized. (PPF: pterygopalatine fossa, ITF: infratemporal fossa).

The ITF is a wide bilateral trapezoid-shaped space, under the floor of the middle cranial fossa and posterior to the maxilla, bounded: (1) laterally by the medial surface of the mandibular ramus, (2) anteriorly by the posterolateral surface of the maxilla (also known as maxillary tuberosity), (3) anteromedially by the lateral pterygoid plate, (4) superiorly by the infratemporal surface of the greater wing of the sphenoidal bone (where the foramen ovale and the foramen spinosum are opened), and by the squamous part of the temporal bone, (5) posteriorly by the tympanic part of the temporal bone and the styloid process (Figure 1 and Figure 2) [19,20]. The remaining surfaces, namely the inferior, the posteromedial, and the superolateral, are opened without any bony boundaries [19,20]. In particular, superolaterally, the ITF is in contiguity with the temporal fossa (the infratemporal crest of the greater wing of the sphenoid marks the border between these two regions), while posteromedially it is separated from the parapharyngeal space by the medial pterygoid muscle and its fascial layer [19]. Indeed, the pre-styloid space (the anterior portion of parapharyngeal space, usually filled with adipose tissue) separates the ITF from the auditory tube and the tensor veli palatini muscle (which lies laterally to the auditory tube), and the levator veli palatini muscle (which is medial and slightly posterior to the tensor veli palatini) [19,20]. The pterygomaxillary fissure, located between the anterior and medial walls of ITF, represents its communication with PPF. ITF is occupied by posterior extensions of the buccal fat pad (i.e., the pterygoid and temporal), muscles, pterygoid venous plexus, branches of the maxillary artery, otic ganglion, lesser petrosal nerve, chorda tympani, the mandibular branch of the trigeminal nerve and its rami [19,20,21].

### 2.2. Muscular and Ligamental Structures

The muscular structures of the ITF are the lateral and medial pterygoid muscles and the tendon of the temporalis muscle [19,20,22]. The lateral pterygoid muscle crosses the upper part of the ITF from anteromedial to posterolateral [19,22]. In most of the cases (61.4–91.1%), it is formed by two heads, one arising from the infratemporal surface of the greater wing of the sphenoidal bone and from the infratemporal crest (superior head) and one from the lateral surface of the lateral pterygoid plate (inferior head) [22,23]. Possible variations of lateral pterygoid muscle with single or triple heads have been reported respectively between 7.7 and 26.7%, and 4.0 and 35.0% of cases [23]. These heads converge in a postero-lateral direction to insert into the pterygoid fovea (a convex space on the neck of the mandible), the articular disc, and the capsule of the temporomandibular joint [22,23,24]. Particularly, the most frequent attachment is at the level of both the condyles and disc and capsule of the temporomandibular joint (55.5%), while insertions only at the level of the condyles (27.8%) or of the disc and capsule (16.7%) were more rarely observed [24]. The pterygoid and temporal extensions of the buccal fat pad, the coronoid process of the mandible, and the insertional tendon of the temporalis muscle are located laterally to the lateral pterygoid muscle, while the medial pterygoid muscle is medial [19,20,21,22]. The medial pterygoid muscle has a quadrangular shape and is located in the lower part of the ITF. It arises with two heads: the superficial one (coming from the lateral surface of the pyramidal process of the palatine bone and the maxillary tuberosity), and the deep one (from the medial surface of the lateral pterygoid plate and from the pterygoid fossa: an osseous depression between the two pterygoid plates) [25,26]. Both heads converge with a postero-lateral direction to attach to the medial surface of the mandibular ramus below the mandibular foramen, at the pterygoid tuberosity, stretching as low as the angle of the mandible [25,26]. The medial pterygoid muscle is connected with the masseter below the inferior margin of the body of the mandible via the mandibular sling [25,26]. The temporalis muscle is inserted onto the coronoid process and temporal crest of the mandible, and it extends outside the ITF in the temporal fossa [22].

The sphenomandibular ligament, one of the temporomandibular joint stabilization structures, is located medially to the condylar process of the mandible, connecting the sphenoidal spine with the lingula of the mandible (spine of Spix), located close to the mandibular foramen [27]. It represents the thicker portion of the interpterygoid fascia, which extends from the base of the skull, lying on the medial surface of the lateral pterygoid muscle and on the lateral of the medial pterygoid muscle, and which is attached to the mandible above the upper border of the insertion of the medial pterygoid muscle up to the neck of the condyle [27]. Together with the lower part of the medial pterygoid muscle, the interpterygoid fascia determines the medial border of a space, called the pterygomandibular space, closed laterally by the articular capsule of the temporomandibular joint and by the mandible, partially occupied inferiorly by the posterior portion of the parotid gland and used as a corridor by the lateral pterygoid nerve and auriculotemporal nerve superiorly and by the inferior alveolar nerve, the maxillary artery, the middle meningeal, and inferior alveolar artery inferiorly [27,28].

### 2.3. Vascular Structures

**Figure 3:**
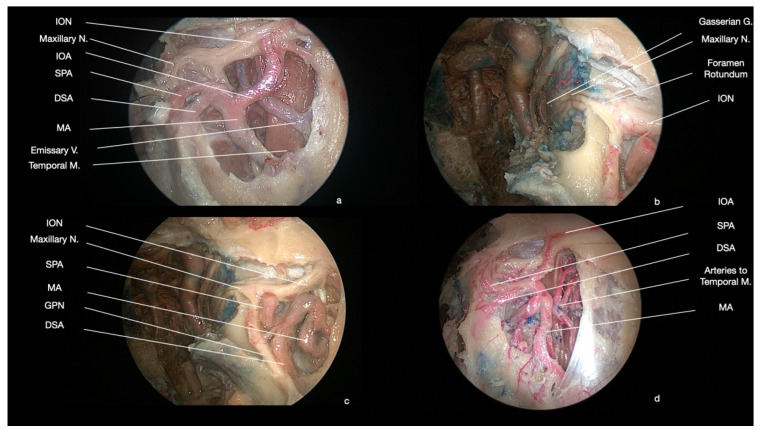
Anatomical endoscopic endonasal dissection (0° degree scope). (**a**) The neurovascular structures of the PPF and ITF are visible, after the removal of the adipose tissue which fills these areas. (**b**,**c**) The maxillary nerve enters the PPF through the foramen rotundum. The pterygopalatine ganglion is located in front of the pterygoid canal, infero-medially to foramen rotundum. (**d**) The maxillary artery courses with an anterior, superior, and medial direction, ending as the infraorbital artery. In this path, it gives origin to multiple branches, which can be identified considering their course and direction. (A: artery, V: vein, M: muscle, N: nerve, G: ganglion, ION: infraorbital nerve, IOA: infraorbital artery, SPA: sphenopalatine artery, DSA: descending palatine artery, MA: maxillary artery, GPN: greater palatine nerve).

**Figure 4:**
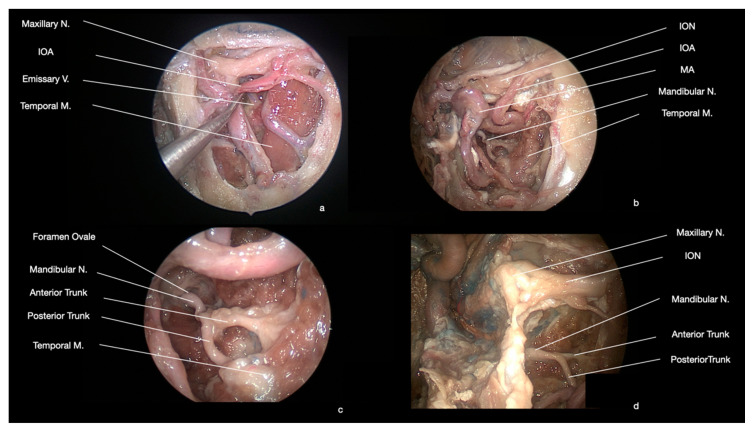
Anatomical endoscopic endonasal dissection (0° degree scope). The mandibular nerve (V3) is contained in the ITF. Immediately after its exit from the foramen ovale, it divides into a smaller anterior and a larger posterior trunk. (A: artery, V: vein, M: muscle, N: nerve, ION: infraorbital nerve, IOA: infraorbital artery, MA: maxillary artery). Anatomica endoscopic endonasal dissection (o° scope). (**a**,**b**)The mandibular nerve (V3) is contained in the ITF. (**c**,**d**) Immediately after its exit from the foramen ovale, it divides into a smaller anterior and a larger posterior trunk.

The structures of the ITF are mainly supplied by the maxillary artery, which originates from the external carotid artery near the posterior border of the condylar process of the mandible (Figure 3 and Figure 4). Its course can be divided into three segments: mandibular, pterygoid, and pterygopalatine [29,30]. The mandibular segment of the maxillary artery passes medially to the condylar process of the mandible and laterally to the sphenomandibular ligament along the inferior border of the lower head of the lateral pterygoid muscle with a horizontal direction [29,30]. In its course, it gives origin to the deep auricular artery (which pierces the anterior aspect of the cartilage of the external acoustic meatus to supply the canal itself and the tympanic membrane), the anterior tympanic artery (which enters into the tympanic cavity through the petrotympanic fissure to supply the mucosa of this region), the middle meningeal artery (which ascends medially to the lateral pterygoid muscle, entering the foramen spinosum, to provide the major supply to dura mater; eventually an accessory meningeal artery can be present, arising separately from the maxillary artery or with a common origin with the middle meningeal artery, directed toward the foramen ovale), and the inferior alveolar artery (which is directed infero-anteriorly, running on the medial wall of the mandibular ramus and entering the mandibular foramen) [29,30].

Afterward, the maxillary artery runs medial to the lateral pterygoid muscle in about 30–45% of cases and lateral in about 55–70% [31]. The pterygoid segment of the maxillary artery gives origin to the deep temporal, the pterygoid and the masseteric arteries (directed toward the homonymous muscles), and the buccal artery (which provides the vascular supply to the buccinator muscle and to mucous membrane and skin of the cheek) [29,30]. The pterygopalatine segment runs in its initial tract between the two heads of the lateral pterygoid muscle, then it enters the PPF through the pterygomaxillary fissure [29,30].

The ITF contains the pterygoid venous plexus, formed by two portions in communication one with each other: a superficial, lesser represented, located between the temporalis and lateral pterygoid muscles, and a deep, usually larger, located between the lateral and medial pterygoid muscles, around the lingual and inferior alveolar nerves, embedded in fat and loose areolar tissue [19]. The deep portion of this plexus is in communication with the foramen lacerum, foramen venosum, cavernous sinuses (through emissary veins passing through the foramen ovale and spinosum), inferior ophthalmic vein (via the inferior orbital fissure), and with the facial vein (by the deep facial vein). It is drained by the maxillary vein, which accompanies the mandibular segment of the maxillary artery, into the retromandibular vein, and finally, into the internal jugular vein [19,20,21].

### 2.4. Nervous Structures

The main neural structure of the ITF is represented by the mandibular nerve (also known, especially in clinical practice, as V3). It passes through the foramen ovale, and within the ITF it gives a first meningeal branch, which turns back through the foramen spinosum into the middle cranial fossa to supply the dura mater [19,28]. Approximately after 5–12 mm from the foramen ovale, medially to the upper head of the lateral pterygoid muscle, the mandibular nerve divides into an anterior and posterior trunk, respectively, of smaller and larger sizes (Figure 4). The otic ganglion lies medially to the mandibular nerve at this level, and it receives the parasympathetic fibers from the lesser petrosal nerve [19,28]. The anterior trunk has a very short path, giving origin to the deep temporal nerves (usually 2 or 3), the masseteric nerve, and the lateral pterygoid nerve (they all provide the motor innervation for homonymous muscles), and to the buccal nerve, (which passes anterolaterally between the two heads of the lateral pterygoid muscle and then laterally to the lower head, to reach the buccinator muscle, where it divides into multiple nerves to provide the sensory supply to the skin of the cheek and to the oral cavity and the visceromotor innervation for local salivatory glands) [19,28]. The posterior trunk is medial to the lateral pterygoid muscle, and it gives off the inferior alveolar, the lingual, the auriculotemporal, the medial pterygoid, the tensor palati, and the tensor tympani nerves (these last three provide the motor innervation for homonymous muscles). In their superior tracts, the lingual and the inferior alveolar nerves run between the lateral and medial pterygoid muscles [19,28]. Afterward, the inferior alveolar nerve passes between the medial pterygoid muscle and the mandible, where it gives origin to two motor branches for the mylohyoid muscle and the anterior belly of the digastric muscle, before entering the mandibular foramen and canal in the medial surface of the mandible [19,28]. Moreover, the lingual nerve passes between the medial pterygoid muscle and the mandible, but then it occupies the gingivo-lingual sulcus [19,28]. Approximately 11 mm after its origin from the posterior trunk, the lingual nerve receives the chorda tympani, a branch of the facial nerve (CN VII), which enters the ITF through the petrotympanic fissure and passes posteromedial to the posterior trunk. In the floor of the mouth, the lingual nerve runs aside to the tongue laterally to the hyoglossus and genioglossus muscles and medially to the sublingual gland [19,28]. The auriculotemporal nerve usually splits into two rami to form a loop encircling the middle meningeal artery, then it continues, passing between the sphenomandibular ligament and the mandible directed toward the parotid gland, which crosses to provide the sensory supply to the skin of temporal region and a part of the auricle including tympanic membrane (outer surface) and a part of the external acoustic meatus [19,28]. It carries on also postganglionic parasympathetic fibers from the otic ganglion to the parotid gland [19,28]. A further nervous structure, present with a very short path into the ITF, is the lesser petrosal nerve, which runs on the floor of the middle cranial fossa, then passes through the foramen petrosum/ovale/lacerum to enter into the ITF, directed toward the otic ganglion, carrying on the preganglionic parasympathetic fibers for the parotid gland [19,28].

## 3. Anatomy of Pterygopalatine Fossa

### 3.1. Boundaries

The PPF is a narrow space, bounded posteriorly by the pterygoid processes, medially by the perpendicular plate of the palatine bone, anteriorly by the maxillary tuberosity, and superiorly by the maxillary surface of the greater wing of the sphenoidal bone (Figure 1 and Figure 2) [28,32,33]. It is open laterally into the ITF through the pterygomaxillary fissure, which continues superiorly with the medial part of the inferior orbital fissure, permitting the direct communication between the orbit and the PPF [28,32,33]. Inferiorly, the fossa has a funnel shape, with an inferior apex, opening into the greater and lesser palatine canals, permitting communication with the oral cavity. In the upper part of the medial wall of the PPF, the sphenopalatine foramen creates a communication between this fossa and the nasal cavity [28,32,33]. The foramen rotundum (opened just below the superior orbital fissure in the posterior wall of PPF) and the pterygoid canal (located infero-medially to the foramen rotudum) provide the communication with the middle cranial fossa [28,32,33]. It presents a further connection, the palatovaginal canal, formed inferiorly by the vaginal process of the body of the sphenoidal bone and superiorly by the antero-inferior wall of sphenoidal sinus, directed toward the posterior part of the roof of the nasopharynx, occupied by the artery of palatovaginal canal. The PPF is filled with adipose tissue (i.e., the pterygopalatine extension of the buccal fat pad), and it contains the maxillary nerve and its branches, the pterygopalatine ganglion, the nerve of the pterygoid canal and the pterygopalatine segment of the maxillary artery, as well as its branches and venous counterparts [28,32,33].

### 3.2. Vascular Structures

The main arterial structure of the PPF is the pterygopalatine segment of the maxillary artery, which passes through the pterygomaxillary fissure [29,30]. It courses with an anterior, superior, and medial direction, ending as infraorbital artery, which enters the orbit via the inferior orbital fissure and continues into a homonymous groove and canal together with the nerve and vein (Figure 3 and Figure 4). As the pterygopalatine segment enters into the PPF, it gives origin to the posterior superior alveolar artery (directed downward to the posterior lateral wall of the maxillary sinus) [29,30]. The remaining branches of this segment arise within the PPF and they are constituted by the recurrent meningeal artery (directed toward the foramen rotundum to enter back into the middle cranial fossa), the descending palatine artery, giving off the greater and lesser palatine arteries (coursing into the greater and lesser palatine canals to supply the hard and soft palate, respectively), the artery of the pterygoid canal (entering the homonymous canal), the pharyngeal artery (running in the palatovaginal canal), which could give origin to the artery of the palatovaginal canal (otherwise directly coming from the maxillary artery) and the sphenopalatine artery (reaching the nasal cavity through the sphenopalatine foramen) (Figure 3) [29,30].

### 3.3. Nervous Structures

The most relevant neural structure of the PPF is the maxillary nerve (also known, especially in clinical practice, as V2), which passes through the foramen rotundum, after the release of a meningeal branch (Figure 3). Inside the PPF, the maxillary nerve immediately gives more branches: the posterior nasal nerves (directed to the pterygopalatine ganglion), the zygomatic nerve, and the posterior superior alveolar nerves [28,32,33]. It is directed anterolaterally, crossing the fossa toward the inferior orbital fissure (Figure 3). The posterior nasal nerves have two components: somatosensory fibers, for the nasal and oral cavities, which enter the ganglion without synapsing and then run into the greater and lesser palatine nerves; and post-ganglionic parasympathetic fibers directed from the ganglion first to the maxillary nerve and then passing into the zygomatic nerve. The zygomatic nerve runs parallel to the infraorbital nerve in the PPF directed toward the inferior orbital fissure [28,32,33]. It enters the orbit and then passes into the zygomatic bone, providing the sensory supply for skin of the temporal and zygomatic regions. In its paths, it gives a communicating branch to the lacrimal nerve, containing the post-ganglionic parasympathetic fibers to the lacrimal gland, originated in the pterygopalatine ganglion. The infraorbital nerve runs within the infraorbital groove and canal, and it gives origin to the middle and anterior superior alveolar nerves [28,32,33]. It ends at the level of the infraorbital foramen, providing several branches innervating the skin of the upper lip, cheek, wing of the nose, and lower eyelid. The superior alveolar nerves run in many tiny canals to reach the dental alveoli of the superior dental arcade. The pterygopalatine ganglion is located in front of the pterygoid canal, infero-medially to the maxillary nerve [28,32,33]. It receives fibers from the autonomic mixed nerve of the pterygoid canal (also known, especially in clinical practice, as the vidian nerve), which is formed by the greater petrosal nerve (carrying on the pre-ganglionic parasympathetic fibers from the facial nerve), and deep petrosal nerve (carrying onpost-ganglionic sympathetic fibers, which passes through the ganglion without synapsis) [28,32,33].

## 4. Tumors of Infratemporal and Pterygopalatine Fossae

ITF and PPF can be involved either by primitive tumors or by the direct tumor invasion from other nearby areas. They can also be the site of metastatization from tumors located in different organs. Among primitive lesions, benign and malignant processes can arise from all the anatomical structures described above [34,35,36]. Although most of these tumors involve both the fossae at the same time, those located medially to the pterygomaxillary fissure are considered PPF lesions, and those laterally as ITF neoplasms.

Juvenile nasopharyngeal angiofibroma (JNA), schwannoma, neurofibroma, cavernoma, pleomorphic adenoma, epithelioid hemangioendothelioma, benign granular cell tumor, and meningioma are the most common benign neoplasms described. Malignant tumors include adenoid cystic carcinoma, chondrosarcoma, osteosarcoma, solitary fibrous tumor (high grade), and rhabdomyosarcomas.

Hematological lesions such as eosinophilic granuloma, diffuse large B-cell lymphoma, and plasmacytoma should be considered in the differential diagnosis [34,35,36]. Invasion of ITF and PFF by bone erosion is reported in inverted papilloma, sinonasal carcinoma, and nasopharyngeal squamous cell carcinoma [34,35,36].

## 5. Pre-Operative Patients’ Management

All patients require a preoperative evaluation with a neurological physical examination, computed tomography (CT) scan, and magnetic resonance imaging (MRI) with intravenous injection of a gadolinium-based contrast agent. Blood bio-humoral assays are also routinely performed, and specific hematological examinations are required in case of a specific clinical suspect.

For tumors protruding in the paranasal sinuses or directly in the nasal cavity, an endoscopic inspection can be performed and, when indicated, can be followed by a biopsy. Furthermore, for all patients suspected to be affected by a vascular lesion, a preoperative angiography with embolization is accomplished 48–72 h before the surgical procedure to reduce the intra- and peri-operative bleeding risks.

## 6. Neuroradiological Imaging

Imaging has a prime role in the management of skull base tumors. The continuous improvement in hardware, acquisition protocols, and advanced image analysis approaches has allowed for a more precise non-invasive assessment of the anatomy and pathological changes of this region. Moreover, coupling advanced imaging with digitally aided surgical techniques, such as image-guided neuro-navigation, has revolutionized the management of skull base tumors. Indeed, accurate preoperative planning with visualization of the vasculature, neural elements, and bone structures within the surgical field and image-guided neuronavigation has increased the efficacy and safety of surgery, ensuring safe resection and sparing vascular and neural injury [37]. Furthermore, imaging allows the surgeon to detect possible anatomical variants, such as variations in the course of the main vessels of the region or the size of the paranasal sinuses, which should be considered in the operative planning to select the best surgical approach to improve patients’ outcome and avoid possible complications [38,39].

Combining the information provided by a CT scan and MRI, the ITF and PPF can be thoroughly investigated. High-resolution CT scan is considered the reference standard for assessing bone structures, whilst MRI is most suited for characterizing lesions, defining their extension, and assessing the presence of perineural spread providing key information for staging, treatment selection, surgical planning, and post-treatment follow-up [40,41].

On a non-contrast-enhanced CT scan (NECT), the normal PPF appears as a hypodense, fat density space. On T_1_-weighted (T_1_W) MR images, the PPF appears hyperintense due to the presence of fat with interposed rounded or linear foci of signal void related to neurovascular structures such as branches of the maxillary artery; mild post-contrast enhancement can be seen also due to small emissary veins [42].

Fat replacement by soft tissue, widening of PPT, and bone erosions are common findings of pathological involvement. Enlargement of the neural foramina and/or fat haziness suggests perineural spread, which is better visualized on fat-saturated contrast-enhanced T_1_W images [43,44]. Post-operative changes within the PPF are almost always visualized as soft tissue, often enhancing, due to scarring hindering the assessment of residual or recurrent disease [45]. The acquisition of an early post-operative baseline scan and serial follow-up imaging allows for the assessment of the stability of post-operative findings, the absence of residual/recurrent local mass lesions, and the visualization of residual/newly developed perineural spread [46].

## 7. Endoscopic Endonasal Approach to Tumors of Infratemporal and Pterygopalatine Fossae

The anatomical relationship at the base of EEA for ITF and PPF lesions is that the anterior boundaries of these two fossae are represented by the posterolateral wall of the maxillary sinus, which can be accessed through an endoscopic anteromedial maxillectomy (Figure 1) [12]. Therefore, thanks to this anterior approach passing through the maxillary sinus, it is possible to achieve direct access to PPF and ITF, avoiding crossing or manipulating any neuro-vascular or muscular structures [12].

In our technique, the patient lies in a semi-sitting position, the thorax slightly elevated (30°), and her/his head tilted toward the first surgeon (20°). Oro-tracheal intubation with the packing of the oropharynx is necessary to prevent blood leakage into the stomach. Surgeries are carried out using HD 2D endoscopes (SPIES, Karl Storz, Tuttlingen, Germany, 4 mm in diameter, 18 cm in length, with 0° and 30° scopes) with a high-definition camera and with the adoption of a neuronavigation (StealthStation S8 MEDTRONIC, Louisville, CO, USA), based on a CT angiogram (CTA), processed through StealthMerge Software (MEDTRONIC, Louisville, CO, USA), and intra-operative EcoDoppler (Mizuho 20 MHz Surgical Doppler System, Mizuho, Tokyo, Japan). Based on the tumor extension, a bi- or mono-nostril (with posterior septostomy) approach was chosen.

The first step of surgery consists of the anteromedial maxillectomy, which requires the complete or partial resection of the inferior nasal concha. This allows the surgeon to identify the natural ostium of the maxillary sinus (semilunar hiatus), which is progressively enlarged to realize the medial maxillectomy (Figure 2). The frontal process of the maxilla, which constitutes the anteromedial wall of the maxillary sinus, is partially removed, usually with a 4 mm diamond high-speed drill (Midas Rex MR7, MEDTRONIC, Louisville, CO, USA). The anterior extension of this drilling depends on the lateral location of the tumor; indeed, more anterior removal of the anteromedial wall of the maxilla permits exposing more lateral portions of these fossae, increasing the instrument’s maneuverability. During this step, the nasolacrimal duct is cut, and eventually, a dacryocystorhinostomy is performed: we suggest performing a sharp cut to reduce the risk of post-operative stenosis with consequent ophthalmological disorders. An ipsilateral complete spheno-ethmoidectomy is associated with medial maxillectomy to increase the maneuverability of the approach.

Then, the perpendicular plate of the palatine bone is drilled out, exposing the sphenopalatine artery, which is coagulated or ligated, preventing any bleeding at this site. Further exposure of PPF and ITF may be facilitated by resecting the medial pterygoid plate and the floor of the sphenoidal sinus, at the level of the pterygoid canal (Figure 2). The identification of this canal gives a relevant anatomical landmark for the internal carotid artery. Indeed, as demonstrated by Kassam et al., it points toward the second genu of the internal carotid artery, at the passage between the intra-petrosal and para-clival tracts (corresponding to the transition between the petrous segment, C2, and cavernous segment, C4, formed by the lacerum segment, C3) [47].

After removal of the posterolateral wall of the maxillary sinus, it is possible to open the periosteum of the ITF and PPF, obtaining access to the pterygopalatine (posteromedially) and infratemporal (posterolateral) regions (Figure 2). From this anterior approach, after the adipose tissue dissection, the most superficial (anterior) structures are represented by the maxillary artery and its branches, which cross the surgical field with an anterior, superior, and medial direction, while the nervous structures lie on a deep plane (Figure 2 and Figure 3). At the level of the superior portion of the PPF, it is possible to dissect the maxillary nerve, which crosses from medial to lateral the surgical field, up to the foramen rotundum, underneath the orbital apex (Figure 3). Moving more laterally and posteriorly, it is possible to expose the medial and lateral pterygoid muscles. Further lateral dissection may identify the masseter and temporalis muscles (Figure 4). Behind the masseter muscle lies the ramus of the mandible, representing the lateral limit of this approach. According to Li et al., the areas of ITF, PPF, and the parapharyngeal space, which can be accessed with this approach, can be divided into five regions: (1) the retromaxillary (between the posterolateral wall of maxillary sinus and medial and lateral pterygoid and temporalis muscles complex, containing the maxillary artery and its branches), (2) the superior interpterygoid (representing the upper part of the ITF and PPF, comprising the superior head of the lateral pterygoid muscle, the mandibular and maxillary nerves and the pterygopalatine and otic ganglia), (3) the inferior interpterygoid (between the inferior head of the lateral pterygoid muscle, the medial pterygoid muscle and temporalis muscle, containing these muscular structures, the branches of the maxillary artery, the anterior and posterior trunk of the mandibular nerve, including the lingual and inferior alveolar nerve), (4) the temporo-masseteric (between the temporalis muscle and zygomatic arch, occupied by adipose tissue), and (5) the tubopharygeal space (corresponding to the upper part of the parapharyngeal space) [48].

Tumors are resected with a bimanual technique. We prefer to fix the endoscope on a holder to use a two surgeons-four hands technique. For benign lesions, the progressively central debulk of the mass is performed and then the tumor is cleaved from the surrounding anatomical structures. Conversely, for malignancies, the lesion is resected in large blocks from the periphery to the center to achieve free surgical margins, confirmed by frozen sections, eventually sacrificing neuro-muscular structures to obtain negative surgical margins. The maxillary artery requires to be legated or clipped as soon as they are identified during the tumor removal, to avoid intra- or post-operative bleedings.

At the end of the surgery, the surgical access in the posterior wall of the maxillary sinus is covered by graft or a pedicled flap of mucoperiosteum from the septum or a nasal concha. Whether the inferior nasal concha was only partially resected at the beginning of surgery, it can be re-sutured and put back in its position. Nasal cavities are packed to prevent post-operative bleeding.

## 8. Postoperative Clinical Management

In the postoperative course, the restoration of spontaneous breathing is obtained at awakening immediately after removal of oro-tracheal intubation. Oral feeding is resumed within 6–8 h after surgery. Nasal packings are removed 48 h later, and patients are discharged from hospital after 3–4 days. We perform a NECT at 6 h after surgery and a post-operative MRI with a gadolinium-based contrast agent within 3 days to assess the degree of tumor resection and detect early surgical complications. If no intra-operative CSF leak occurred, the patients could start a cautions mobilization the same day of surgery; otherwise, 3 days of bed rest are prescribed with antithrombotic prophylaxis.

Follow-up consisted of an endoscopic endonasal medication performed within 30 days from surgery. MRI with gadolinium-based contrast agent and a neurological evaluation were repeated at 3 months. Then, clinical and neuroradiological follow-up was performed at regular intervals of 6–12 months, depending on the pathological diagnosis. For malignancies, complementary therapies (radiotherapy and/or chemotherapy) were performed based on the histological characterization of the tumor [49].

## 9. Illustrative Cases

### 9.1. Case 1

**Figure 5:**
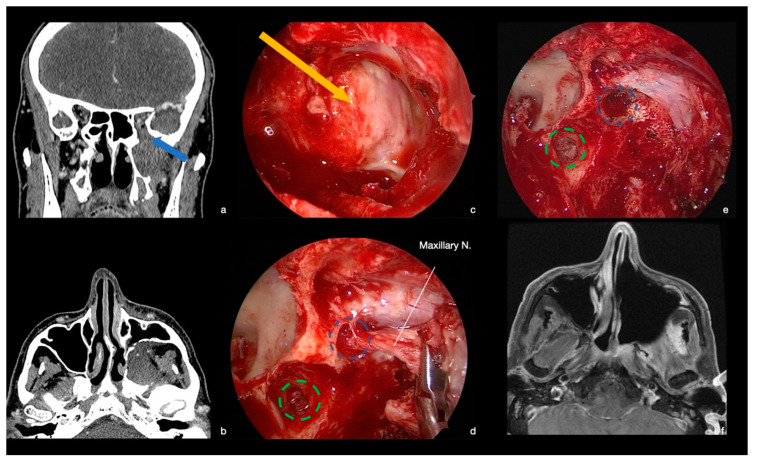
CT scan, MRI, and intra-operative endoscopic images (0° scope). (**a**,**b**) Coronal and axial NECT showing the PPF mass. An enlarged foramen rotundum, due to tumor infiltration of the maxillary nerve was observed (blue arrow). (**c**) Intra-operative image. After medial maxillectomy, at the posterior wall of the maxillary sinus, it was possible to observe a bulge directed anteriorly due to the presence of the mass in the PPF (yellow arrow). The infra-orbital nerve was evident in the roof of the maxillary sinus. (**d**) Intra-operative image. The maxillary nerve resulted infiltrated by the tumor and was resected up the foramen rotundum (blue circle), while the vidian nerve had been already resected at the opening of pterygoid canal (green circle). (**e**) Intra-operative image. At the end of tumor removal, no tumor remnants were observed and foramen rotundum (blue circle) and opening of pterygoid canal (green circle) are visible. (**f**) MRI. Axial slice. T1 w-sequence with contrast agent. Radical tumor resection with no surgical complications was confirmed.

A 53-year-old man came to our attention for the progressive onset of paraesthesiae on the left territory of the maxillary nerve for a few months. A CT scan showed an isodense mass in the left PPF, confirmed by the following MRI, which demonstrated a tumor hypointense in T1 w-sequences, hyperintense in T2 w-sequences, and inhomogeneously enhancing after contrast agent (Figure 5). Past medical history was unremarkable. At physical examination, hypoesthesia in the left V2 territory was noted.

The patient underwent an EEA for the PPF tumor through a transmaxillary-pterygoid approach. After removal of the posterior wall of the maxillary sinus, the tumor was identified. A frozen section was positive for the diagnosis of adenoid cystic carcinoma. The lesion was initially debulked, then it was removed in large blocks from the periphery to the central portion. The frozen sections from the maxillary nerve demonstrated neoplastic infiltration up to the foramen rotundum, and the nerve was consequently resected at this level (Figure 5). Similarly, the nerve of the pterygoid canal was sacrificed for its tumoral infiltration. At the end, a radical resection (R0) was achieved (Figure 5). The histological evaluation showed a slow-growing tumor with epithelial and myoepithelial neoplastic cells that had a prevalent cribriform pattern, characterized by nests of tumor cells interrupted by sharply punched-out spaces filled with basophilic matrix. Perineural invasion was confirmed. Immunohistochemical staining for KIT (CD117) was restricted to inner epithelial cells and p63 and SMA to peripheral myoepithelial cells. The final diagnosis was adenoid cystic carcinoma.

The postoperative course was unremarkable. The patient was awakened at the end of surgery and oral feeding was resumed the same day. He was discharged 3 days later with a hypoesthesia in the V2 territory, which was unchanged in comparison to the pre-operative examination. Post-operative MRI confirmed the complete tumor resection (Figure 4). He performed radiation therapy with heavy particles (protons) in the following months. At follow-up (62 months), no recurrence was observed, and the patient is in good general conditions.

### 9.2. Case 2

**Figure 6:**
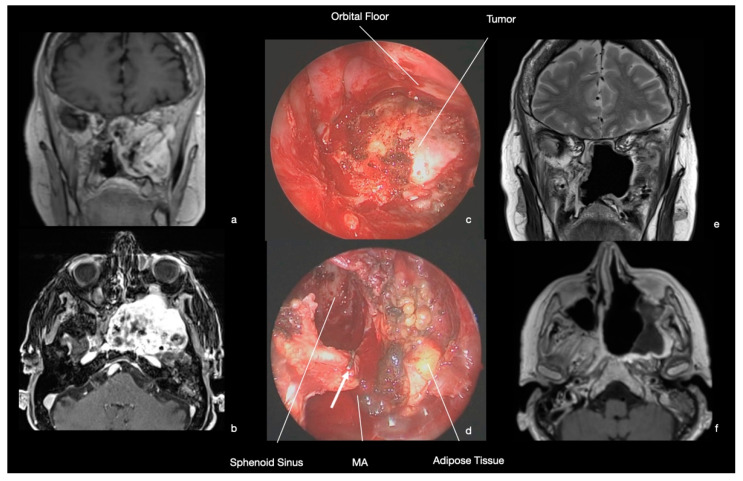
MRI and intraoperative endoscopic images (0° scope). (**a**,**b**) MRI. T1 w-sequence with contrast agent. A large mass extending in the left PPF and ITF was demonstrated. The floor of the orbit was pushed superiorly, with no suspect of periorbit infiltration. (**c**) Intra-operative image. After medial maxillectomy and resection of the posterior wall of maxillary sinus, the soft tumor was progressively resected. It was possible to cleave the lesion from the orbital floor. (**d**) Intra-operative image. At the end of the surgery, the tumor was completely resected with preservation of PPF anatomical structures. The maxillary artery (MA) has been identified and clipped (white arrow) during the tumor removal. (**e**,**f**) MRI. Coronal and axial view. T2 w-sequence and T1 w-sequence with contrast agent. Radical tumor resection was confirmed with no surgical complications.

A 69-year-old female was referred to our center for the progression of a large PPF and ITF tumor. The patient had a long history of hypoesthesia with paranesthesia in the territory of the left mandibular nerve and masticatory difficulties. After an MRI, showing the skull base lesion, a radiosurgical treatment was performed. During the follow-up, symptoms worsened, and at neuroimaging, an increase in the dimension of the mass was confirmed. At MRI, the tumor was hypointense in T1 w-sequences and hyperintense in T2-w with inhomogeneous contrast enhancement (Figure 6). At physical examination, left V3 hypoesthesia was confirmed.

A transmaxillary-pterygoid endoscopic approach was chosen. After the resection of the posterior wall of the maxillary sinus, a mass with a soft consistency was observed. A central debulking was performed and the tumor was progressively cleaved from pterygoid muscles, dura of the middle cranial fossa, and floor of the orbit. The maxillary artery was clipped during tumor resection (Figure 6). The histological examination showed a spindle cell tumor composed nearly entirely of well-differentiated Schwann cells compatible with conventional schwannoma (CNS WHO grade 1).

She was awakened at the end of the surgery, with immediate restoration of spontaneous breathing and recovery of oral feeding on the same day. The patient was discharged home 3 days later with a hypoesthesia in the V3 territory, which was unchanged in comparison to pre-operative examination. Post-operative MRI showed the radical tumor resection (Figure 5). At 41 months follow-up, no recurrence was observed, and the patient is in good general condition.

### 9.3. Case 3

**Figure 7:**
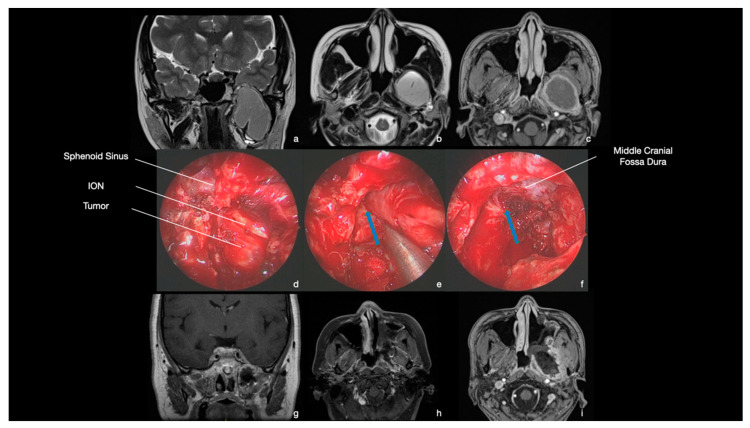
MRI and intraoperative endoscopic images (0° scope). (**a**–**c**) MRI. Coronal and axial T2 w-sequence and T1 w-sequence with contrast agent. A large tumor was observed in the left PPF and ITF. (**d**) Intra-operative image. After medial maxillectomy, the posterior wall of the maxillary sinus is removed, and the tumor is exposed. The mass was of firm consistency and diffusely bleeding. (**e**) Intra-operative image. Tumor was progressively resected, and the mandibular nerve was identified and anatomically preserved until the foramen ovale (blue arrow). The mass was cleaved from middle cranial fossa dura, without any CSF leak. (**f**) Intra-operative image. At the end of the surgery, the tumor was radically removed, with preservation of anatomical structures. (**g**–**i**) MRI. Coronal and axial T1 w-sequence with contrast agent. Complete tumor removal was demonstrated.

A 28-year-old female presented hypoesthesia in the territory of the left mandibular nerve and masticatory difficulties for a few months. Medical history was unremarkable. MRI demonstrated an ITF and PPF tumor, which was hypointense in T1 w-sequences and hyperintense in T2-w with a peripheral contrast enhancement (Figure 7). At physical examination, left V3 hypoesthesia was confirmed.

The patient underwent a transmaxillary-pterygoid endoscopic approach. After the resection of the posterior wall of the maxillary sinus, a firm bleeding tumor was observed. Tumor resection was performed, starting with a central debulking. Afterward, the mass was progressively cleaved from the pterygoid and temporalis muscles and from the dura of fossa media without any intra-operative CSF leak. Resection resulted in radical preservation of the anatomical structures (Figure 7). Histological diagnosis showed the proliferation of epithelioid cells forming syncytia-like lobules, with some nuclei appearing to have nuclear holes and pseudoinclusions. Some whorls and psammoma bodies were present. The nuclei of the neoplastic cells expressed the progesterone receptor. Besides, SSTR2A was expressed strongly and diffusely in the neoplastic cells. The proliferation index evaluated with Ki-67 was <3%. The mitotic account was 1 mitosis on 10 HPF. The final diagnosis was meningothelial meningioma (CNS WHO grade 1).

The post-operative course was unremarkable, with awakening and restoration of spontaneous breathing at the end of surgery and recovery of oral feeding on the same day. The patient was discharged home 3 days later with unchanged hypoesthesia than the pre-operative examination. The post-operative MRI showed complete tumor resection (Figure 7). At 38 months follow-up, no recurrence was observed.

## 10. Discussion

The EEA is undoubtedly becoming one of the most relevant approaches for a majority of lesions involving the nose and paranasal sinuses, and for a great number of median and paramedian skull base tumors [50]. The main advantage of this approach is its low morbidity, especially in comparison to other open external approaches, not needing any skin incisions or soft tissue disruption, and with a reduced risk of post-operative neurological deficits [50]. However, since their paramedian location and complexity, ITF and PPF have been only recently considered amenable for EEA and currently, the IT approaches are still being preferred in many Institutions [1,2,3,4].

Although the use of a transmaxillary route for ITF and PPF lesions has been suggested since 1858, the lack of an adequate visualization system along such a long and deep corridor has prevented its use for decades [10,51]. It has been the visualization power and the versatility in the paramedian extension of the endonasal route given by the endoscope that has permitted the re-discovery of this transmaxillary approach [10]. Its first application was in endoscopic-assisted procedures, performed through the traditional external approaches, and only in the late 1990s, Klossek et al. and Mitskavich et al. reported the complete endoscopic endonasal resection, respectively, of a neuroma and of a JNA of the PPF [52,53,54]. Nevertheless, these authors observed that the lack of dedicated surgical instrumentation for such a paramedian transnasal corridor represented an obstacle to the development of this route at that time [52,53].

For these reasons, limited attempts to remove endoscopically tumors of ITF and PPF have been performed in the following years [55]. It is only after the report by Bolger et al., which outlined the good results of the endoscopic transpterygoid approach for the treatment of an encephalocele of the middle cranial fossa located laterally to the foramen rotundum, that the technical feasibility of this route for ITF and PPF has been confirmed [56]. Indeed, these authors demonstrated that the EEA could permit reaching the lateral sphenoidal recess with high maneuverability and a favorable angle of attack, supporting the possible extension of this route also for ITF and PPF tumors [56]. Subsequently, a large number of surgical series have been reported, demonstrating satisfactory surgical results of the transmaxillary-pterygoid approach for the treatment of many skull base tumors, involving or extending to the ITF and PPF, such as schwannomas, meningiomas, chordomas, chondrosarcomas, or other middle cranial fossa lesions [1,2,3,4,14,15,16,17,18]. For such lesions, it should be considered that their very lateral extension, particularly inferiorly to the maxillary sinus floor toward the angle of the mandible, can still represent a caveat, increasing the difficulties of the EEA [48]. Moreover, vascular tumors, such as selected cases of JNAs or hemangiopericytomas, resulted in being suitable for this approach, eventually with preliminary embolization, to reduce intra-operative bleeding [1,2,3,4,14,15,16,17,18,57]. Following this increasing experience, EEA has been adopted also for selected cases of ITF and PPF malignancies, which were previously considered one of its limits, since the need for a complete resection with negative margins [1,2,3,4,14,15,16,17,18]. Indeed, it has been suggested that en-bloc resection is rarely possible for a malignant tumor of this region also with external approaches, and that because EEA can effectively manage the tumor infiltration, as a perineural spread or dural involvement, it would permit achieving the radical resection of these neoplasms [3,58]. However, it should be considered that the lack of formal control of both the internal carotid artery and internal jugular vein, especially for malignancies or vascular lesions infiltrating the parapharyngeal space or encasing or in close relationship with these vascular structures, represents a limit of this approach [1,2,3,4,14,15,16,17,18,48]. Similarly, the tumoral extension to other surrounding regions not reachable with the EEA requires combining this route with other approaches, such as transcranial, temporal, transorbital, or others [1,2,3,4,14,15,16,17,18,48].

An important requisite to approach the ITF and PPF by an endoscopic transmaxillary-pterygoid route is the anteromedial maxillectomy, which permits a great exposure of the most lateral portion of the posterolateral wall of the maxillary sinus and consequently of the most lateral portion of these fossae. An alternative to the anteromedial maxillectomy is represented by the Caldwell–Luc transmaxillary approach, as proposed by Theodosopoulos et al., to reduce the risks of cosmetic deformity and avoid the inferior nasal concha removal with less disruption of the nasal function [59]. However, complications related to the anteromedial maxillectomy have not been significantly reported in the literature [1,2,3,4,14,15,16,17,18]. Moreover, we have recently started to avoid completely cutting the inferior nasal concha, preferring a partial resection, followed by re-suturing at the end of the surgery, furtherly reducing the post-operative nasal discomfort. Moreover, the anteromedial maxillectomy has demonstrated greater surgical freedom and consequently increased instrument maneuverability in comparison also to prelacrimal approaches [60].

Moreover, EEA for ITF and PPF requires a long training curve, and it should be considered an advanced procedure, to be performed in dedicated referral centers [61]. We strongly suggest a careful selection of patients based on pre-operative neuro-imaging and clinical features. Furthermore, given the multidisciplinary nature of these lesions, involving neurosurgeons, ENT, maxillo-facial surgeons, interventional radiologists, oncologists, radiotherapists, and specialists in palliative care, any surgical treatment should be carefully planned and discussed in dedicated skull base boards.

## 11. Conclusions

The EEA is providing an excellent visualization, angle of attack, and working space for selected ITF and PPF benign and malignant tumors. Its main advantages are the reduced transient and permanent morbidity with a low neurological deficits rate and absence of craniofacial scars. It is routinely adopted for primary tumors, but also for lesions deriving from nearby surrounding areas. It can be combined with other open or minimally invasive skull base approaches, such as transcranial, temporal, transorbital, or other routes, to manage also more complex tumors. Normally, it is well tolerated by patients, with quick patient recovery, reduced post-operative discomfort or pain, and, thanks to fast resuming of patients’ normal life activities, it would permit an early access to complementary treatments, when necessary.

Its main limits are represented by the long training curve and by the need for excellent knowledge of the anatomy of these areas, combined with the use of advanced technological devices, to avoid injuries to nervous and vascular structures, whose formal proximal control, especially for vascular or malignant lesions, remains the main limit of this route.

Careful pre- and post-operative patient management is of primary importance, and each treatment should be carefully planned and discussed in dedicated multidisciplinary skull base boards.

## Data Availability

Not applicable.
